# Pathophysiology and Treatment of the No-Reflow Phenomenon in ST-Segment Elevation Myocardial Infarction: Focus on Low-Dose Fibrinolysis during Primary Percutaneous Intervention

**DOI:** 10.31083/j.rcm2412365

**Published:** 2023-12-25

**Authors:** Francesco Pelliccia, Giampaolo Niccoli, Marco Zimarino, Giuseppe Andò, Italo Porto, Paolo Calabrò, Salvatore De Rosa, Felice Gragnano, Raffaele Piccolo, Elisabetta Moscarella, Enrico Fabris, Rocco Antonio Montone, Carmen Spaccarotella, Ciro Indolfi, Gianfranco Sinagra, Pasquale Perrone Filardi, Working Group of Interventional Cardiology of the Italian Society of Cardiology

**Affiliations:** ^1^Department of Cardiovascular Sciences, University Sapienza, 00185 Rome, Italy; ^2^Department of Medicine and Surgery, University of Parma, 43125 Parma, Italy; ^3^Department of Neuroscience, Imaging and Clinical Sciences, ‘G. D'Annunzio' University of Chieti-Pescara, 66100 Chieti, Italy; ^4^Department of Clinical and Experimental Medicine, University of Messina, 98124 Messina, Italy; ^5^Department of Internal Medicine and Specialties, University of Genoa, 16132 Genova, Italy; ^6^Cardiology Unit, Cardiothoracic and Vascular Department (DICATOV) IRCCS, Ospedale Policlinico San Martino, 16132 Genoa, Italy; ^7^Department of Translational Medical Sciences, University of Campania “Luigi Vanvitelli”, 81100 Caserta, Italy; ^8^Division of Clinical Cardiology, A.O.R.N. “Sant'Anna e San Sebastiano”, 81100 Caserta, Italy; ^9^Division of Cardiology, Department of Medical and Surgical Sciences, Magna Graecia University of Catanzaro, 88100 Catanzaro, Italy; ^10^Department of Advanced Biomedical Sciences, University of Naples Federico II, 80131 Naples, Italy; ^11^Cardiothoracovascular Department, Azienda Sanitaria Universitaria Giuliano Isontina (ASUGI), University of Trieste, 34148 Trieste, Italy; ^12^Department of Cardiovascular Medicine, Fondazione Policlinico Universitario A. Gemelli IRCCS, 00168 Rome, Italy

**Keywords:** infarct-related artery, microvascular obstruction, no reflow, percutaneous coronary intervention, ST-elevation myocardial infarction

## Abstract

Primary percutaneous coronary intervention (PCI) is the current class I therapeutic approach to treat acute ST-elevation myocardial infarction (STEMI). While primary PCI can restore adequate flow in the infarcted artery in the majority of cases, some patients experience the ‘no-reflow’ phenomenon, i.e., an abnormal myocardial reperfusion occurring even after the occluded coronary artery has been opened. No-reflow occurs when microvascular obstruction arises from embolization of thrombus or components of the atheromatous plaques. These embolic materials travel downstream within the infarct-related artery at time of primary PCI, leading to compromised blood flow. Currently, no expert consensus documents exist to outline an optimal strategy to prevent or treat no-reflow. Interventional cardiologists frequently employ intracoronary adenosine, calcium channel blockers, nicorandil, nitroprusside or glycoprotein IIb/IIIa inhibitors. However, evidence suggests that these interventions consistently enhance myocardial blood flow in only a specific subset of patients experiencing no-reflow. A recent and innovative therapeutic approach gaining attention is low-dose fibrinolysis during primary PCI, which offers the potential to augment coronary flow post-myocardial revascularization.

## 1. Introduction

Acute ST-elevation myocardial infarction (STEMI) is a severe clinical presentation of coronary artery disease (CAD). Interestingly, STEMI prevalence is disproportionately higher in low and middle-income countries when compared to high-income states [1]. Additionally, STEMI treatment is particularly expensive, especially in the United States [2]. Coronary revascularization, and percutaneous coronary intervention (PCI) stand out as significant contributors to the cost [3]. Primary PCI is a class I therapeutic approach to manage STEMI, as stated by the recommendations of the European Society of Cardiology [4]. This strategy has proven highly effective in restoring normal coronary blood flow in the epicardial vessels for over 95% of patients, significantly improving their prognosis. Despite the success of revascularization, nearly 60% of patients with STEMI encounter suboptimal coronary reperfusion, resulting in slow, incomplete, or even absent coronary flow [4]. This phenomenon, known as ‘no-reflow’, is attributed to structural and functional changes in the coronary microcirculation, hindering the reperfusion of previously ischemic myocardial regions after the occluded coronary artery is reopened [5]. The no-reflow phenomenon, whether assessed non-invasively or invasively during primary PCI, has been associated with various adverse outcomes, including ventricular arrhythmias, left ventricular dysfunction, impaired ventricular remodeling, myocardial re-infarction, and increased mortality [6, 7].

In this article, we review the pathophysiology and treatment of the no-reflow phenomenon in patients with STEMI. Notably, Adjedj *et al*. [8] provided an elegant description of no-reflow management, offering valuable insights to prevent the complication as well as adapting therapeutics to limit myocardial damage when no-reflow occurs. Furthermore, Alexiou *et al*. [9] conducted a meta-analysis, which demonstrated that intracoronary thrombolysis was associated with improved major adverse cardiac events and myocardial microcirculation in STEMI patients undergoing primary PCI. With this background, we will discuss standard pharmacologic options currently applied in the catheterization laboratory to treat no-reflow and highlight novel therapeutic options that have been the focus of several recent investigations.

## 2. Pathophysiology of Coronary Microvascular Obstruction

**Fig. 1.**
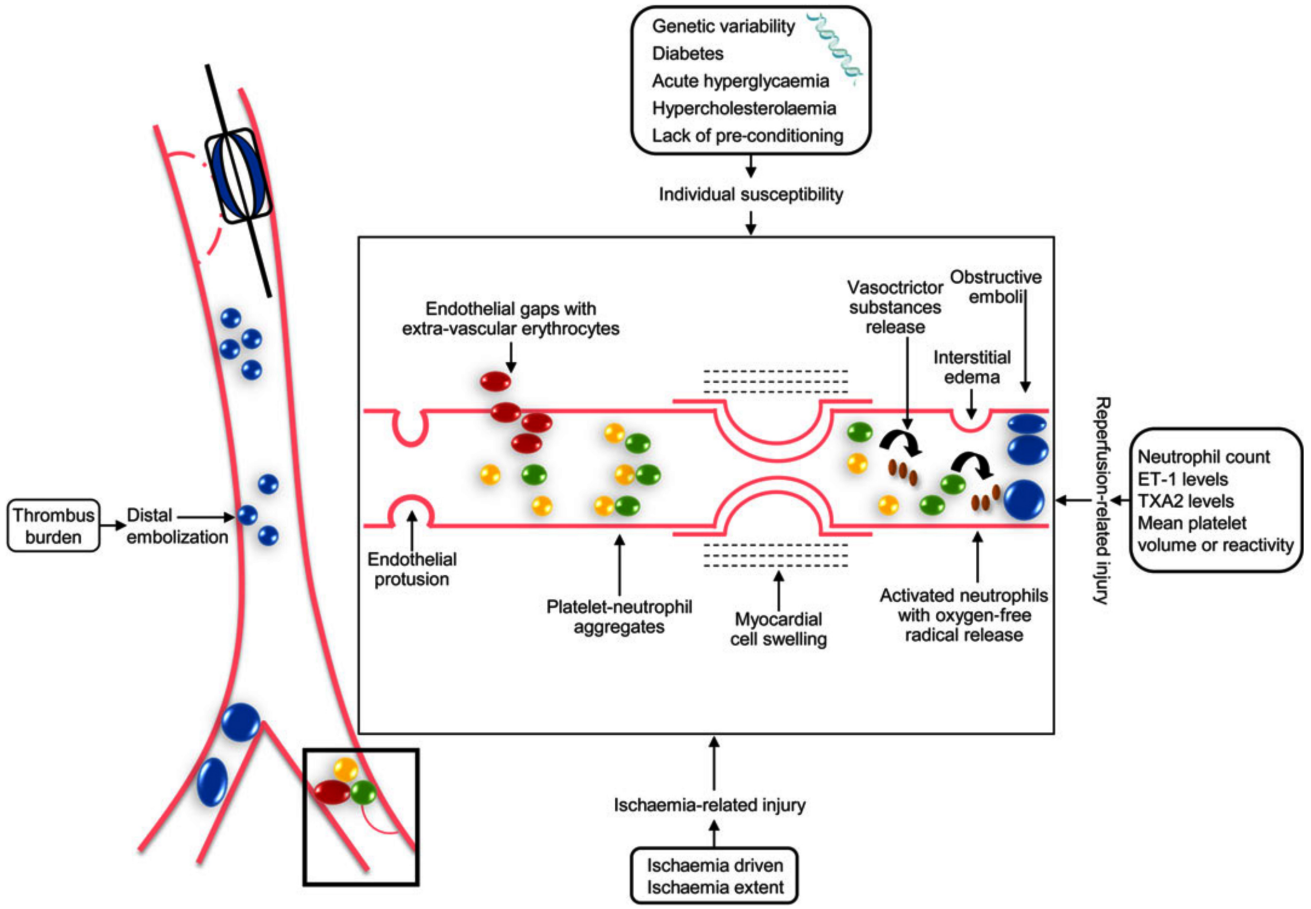
**Interacting mechanisms involved in the pathogenesis of coronary microvascular obstruction in humans (Reproduced with permission by Niccoli *et al*. [10] European Heart Journal (2016) 37, 1024–1033)**. ET-1, endothelin-1; TAX2, thromboxane A2.

Multiple interacting mechanisms have been implicated in the pathogenesis of coronary microvascular obstruction, including reperfusion injury, thrombus embolization, and genetic or pre-existing susceptibility to injury of the microcirculation (Fig. 1) [10, 11]. Reperfusion injury occurs when blood flow is abruptly restored in an infarct-related artery, which in turn causes an influx of neutrophils and triggers the production of free oxygen radicals, proteolytic enzymes and cytokines. These pathophysiological abnormalities can have multiple irreversible consequences, including microvascular obstruction [12, 13, 14]. In addition, distal embolization of an atherosclerotic lesion resulting from primary PCI might increase vascular resistance and trigger an increase of circulating levels of inflammatory agents and substances with vasoconstrictive properties [15, 16, 17]. Moreover, no-reflow has been associated with pre-existing endothelial dysfunction as well as to genetic mutations [6, 10, 14]. Some of the mutations include adenosine receptor polymorphisms and endothelial ion channels, which have the potential to increase the vulnerability to either small vessel dysfunction and no-reflow [18, 19, 20].

## 3. Pathology of No-Reflow Phenomenon

**Fig. 2.**
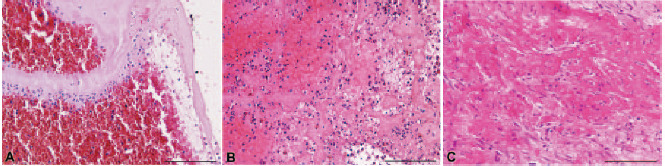
**Histological spectrum of thrombus characteristics at time of primary percutaneous coronary intervention**. (A) The image displays a fresh thrombus with leukocytes, platelets, erythrocytes, and fibrin. (B) Here a stabilized thrombus is visible with lytic and organized areas, indicating that the thrombus is 1 to 2 days old. (C) In this picture, an organized thrombus is shown, demonstrating collagen deposition in the homogenized thrombus, indicating an older thrombus. Hematoxylin and eosin stains were used for tissue visualization. Bars = 150 µm (Reproduced with permission by Kramer *et al*. [21] *Circulation*. 2008;118:1810–1816).

In recent years, pathology studies have provided insight into the possible mechanisms underlying no-reflow in case after primary PCI for STEMI. Histologic investigations have revealed that thrombotic material at the site of a coronary artery occlusion includes not only fibrin, platelets, erythrocytes, and leucocytes, but also lytic and organized areas of thrombus [21]. These findings suggest that an abrupt coronary occlusion may be often the final step of several non-occlusive atherothrombotic events that occur days or weeks earlier (Fig. 2, Ref. [21]) [20]. Indeed, thrombi with different characteristics (i.e., fresh vs organized) result in different types of microvascular obstruction at the time of primary PCI in patients with STEMI, necessitating different treatments (i.e., antiplatelet agents vs fibrinolytic drugs). The presence of a stabilized older thrombus, therefore, might explain why several pharmacologic (i.e., antiplatelet drugs, including clopidogrel, ticagrelor, prasugrel, glycoprotein IIb/IIIa inhibitors and bivalirudin) or mechanical strategies (thrombectomy with aspiration) tested in recent years to optimize primary PCI have often yielded poor results [22, 23, 24].

## 4. Current Management of No-Reflow Phenomenon

Despite extensive investigation into no-reflow over the last decade, the current treatment of this complication is still based on the intracoronary administration of pharmacologic agents with varying mechanisms of action. Indeed, several cardioactive agents, including beta-blockers, calcium channel blockers, adenosine, and sodium nitroprusside, as well as antiplatelet drugs such as cangrelor and glycoprotein IIb/IIIa inhibitors currently used in treatment [25].

### 4.1 Conventional Pharmacologic Approach to No-Reflow

Beta-blockers have the potential to protect cardiomyocytes and limit infarct extension. In the first of two major trials of STEMI patients undergoing primary PCI, EARLY-BAMI (Early-Beta blocker Administration before reperfusion primary PCI in patients with ST-elevation Myocardial Infarction) assessed the effects of two 5-mg boluses of metoprolol. The first 5-mg bolus was administered in the ambulance and the second 5-mg bolus was given in the catheterization laboratory [26]. The results revealed no significant differences between the metoprolol and placebo groups in terms of infarct size, as assessed by percent of left ventricular delayed enhancement on cardiac magnetic resonance imaging (15.3 ± 11.0% vs 14.9 ± 11.5%; *p* = 0.616).

In contrast, the METOCARD-CNIC (Effect of Metoprolol in Cardioprotection During an Acute Myocardial Infarction) compared the effects of intravenous metoprolol (15 mg) with placebo [27]. The trial evaluated infarct size, as assessed through magnetic resonance imaging (MRI), and found that it was smaller after intravenous metoprolol administration compared to the control group (25.6 ± 15.3 g vs. 32.0 ± 22.2 g; 95% confidence interval [CI], –11.39 to –1.78; *p* = 0.012) [27]. The size reduction was accompanied by an increase in left ventricular ejection fraction with metoprolol, as observed by MRI, one week after STEMI (46.1 ± 9.3% vs 43.4 ± 10.4% in the controls; unadjusted difference 2.74; 95% CI: 0.13 to 5.35; *p* = 0.039) [27]. In a related study, Pizarro *et al*. [28] reported that patients treated with metoprolol maintained a significantly higher left ventricular ejection fraction at the 6-month evaluation compared to the control group (48.7 ± 10.0% vs. 45.0 ± 11.7%, unadjusted difference 3.67; 95% CI: 0.64–6.71; *p* = 0.018). At a median follow-up of 2 years, the occurrence of major adverse cardiac events (i.e., a composite of death, heart failure admission, reinfarction, and malignant arrhythmias) was 10.8% in the metoprolol group compared to 18.3% in the controls (adjusted hazard ratio [HR]: 0.55; 95% CI: 0.26 to 1.04; *p*= 0.065) [28]. Furthermore, there was evidence of a 40% reduction in the size of microvascular obstruction in patients treated with metoprolol [29]. The different results observed between the EARLY-BAMI and METOCARD-CNIC trials can likely be attributed to variations in patient selection, study design, and statistical power. Consequently, further studies are necessary to assess the potential benefits of intravenous metoprolol in STEMI patients undergoing primary PCI.

Calcium channel blockers might affect no-reflow through diverse mechanisms. By targeting L-type calcium channels, these drugs can regulate the influx of calcium into vascular smooth muscle, cardiac myocytes, and cardiac nodal tissue [30]. By blocking calcium entry into the cell, they can induce vascular smooth muscle relaxation, resulting in vasodilation and reduced myocardial force generation, leading to decreased contractility, negative chronotropy, and consequently a lower heart rate [31]. The effects of verapamil, diltiazem and nicardipine on no-reflow have been extensively investigated and well-documented [30, 31, 32]. A meta-analysis of 5 trials with a total of 325 participants randomized to receive verapamil/diltiazem (n = 162) or control therapy (n =163) demonstrated that intracoronary verapamil/diltiazem significantly decreased the occurrence of the coronary no reflow phenomenon (relative risk [RR]: 0.3, 95% CI: 0.16 to 0.57; *p* = 0.0002, *$I^{2}$* = 4%) [9]. In a retrospective analysis of 72 patients who received intracoronary nicardipine during PCI, no-reflow was successfully reversed with complete restoration of thrombolysis in myocardial infarction (TIMI) 3 flow in 71 of 72 patients (98.6%) [32]. TIMI flow grade improved from 1.65 ± 0.53 at baseline to 2.97 ± 0.24 after nicardipine (*p* < 0.001), and TIMI frame count decreased from 57 ± 40 at time of no-reflow to 15 ± 12 after nicardipine (*p* < 0.001) [32]. Intracoronary nicardipine provides additional benefits in preventing no-reflow when combined with rotational atherectomy [31]. In a prospective registry, 150 of 155 patients (96.7%), and 175 of 181 treated vessels (96.6%) showing TIMI 3 flow at the completion of the combined treatment [33]. Overall, the final TIMI score worsened in 4 patients (2%), was unchanged in 121 patients (78%), and improved in 30 patients (19%) compared to baseline [33].

Adenosine is frequently utilized in catheterization laboratories to treat no-reflow, offering benefits including vasodilation of the coronary microcirculation through smooth muscle relaxation. The REOPEN-AMI (Intracoronary Nitroprusside Versus Adenosine in Acute Myocardial Infarction) trial demonstrated that adenosine significantly improved microvascular obstruction [34]. Notably, a greater frequency of ST-segment resolution (>70%) was observed 90 minutes after PCI (measured by 12-lead electrocardiogram) in patients treated with adenosine compared to those receiving sodium nitroprusside or saline infusion (71% vs. 54% and 51%, respectively; *p* = 0.009 and *p* = 0.75) [34]. Moreover, this improvement was associated with a decrease in major cardiovascular events and a favorable cardiac remodeling during the 1-year follow-up [34].

These findings differed from the results of previous studies, including the AMISTAD (Acute Myocardial Infarction Study of Adenosine) trial [35] and AMISTAD II trial [36] . The AMISTAD trial found a 67% relative reduction in infarct size in patients with anterior infarction (15% in the adenosine group vs 45.5% in the placebo group), with no decrease in patients who suffered from an infarction in different myocardial regions (11.5% for both groups) [35]. The AMISTAD II trial found no difference in the primary endpoint (a combination of heart failure, first re-hospitalization, or cardiac and non-cardiac events during the 6-month follow-up) between the placebo group (17.9%) and groups receiving different doses of adenosine (16.3%) [36]. Furthermore, the REFLO-STEMI (Reperfusion Facilitated by Local Adjunctive Therapy in ST-Elevation Myocardial Infarction) trial also reported no significant difference in infarct size between the adenosine treatment group (median: 10.1, interquartile range: 4.7–16.2), the sodium nitroprusside group (median: 10.0, interquartile range: 4.2–15.8), and control (median: 8.3, interquartile range: 1.9–14.0) (*p* = 0.062 and *p* = 0.160 vs controls, respectively) [37]. These findings are consistent with the results of a recent meta-analysis, which demonstrated that adenosine treatment leads to a higher frequency of atrio-ventricular blocks and ventricular arrhythmias in patients with acute coronary syndrome compared to placebo [38].

Sodium nitroprusside is a pro-drug that is metabolized to nitric oxide, the active form that affects both circulation and platelets. Nitric oxide exhibits potent vasodilation in both coronary and peripheral microcirculation while also exerting antiplatelet activity through inhibition of platelet aggregation [39, 40]. Notably, the use of sodium nitroprusside has been linked to a reduction in the frequency of outcomes, including an increase in TIMI frame count, faster improvement of ST-segment upsloping, and an improved left ventricular function [41]. At present, there is a lack of clinical data supporting the use of sodium nitroprusside in no-reflow prevention, thus further studies are needed to address this issue [42].

Epinephrine has emerged as another promising pharmacological agent for no-reflow [43, 44], particularly in cases unresponsive to other treatments. Specifically, the results of the RESTORE trial, a multicenter research aimed at evaluating the safety and efficacy of epinephrine in post-STEMI no-reflow have been recently published [45]. The authors of the study found that administration of epinephrine through the intracoronary route was associated with a significant improvement in coronary blood flow (TIMI 3: 28.6%, TIMI 2: 64.3%, TIMI 1: 7.1%, and TIMI 0: 0%), in comparison to the patients who received traditional drugs (TIMI 3: 18.8%, TIMI 2: 12.5%, TIMI 1: 37.5%, and TIMI 0: 31.3%) (*p* value between groups = 0.004) [45]. Subsequently, the COAR (Comparison of Intracoronary Epinephrine and Adenosine for No-Reflow) trial, a randomized trial comparing epinephrine and adenosine, has demonstrated a significantly greater improvement in no-reflow with epinephrine, as shown by higher TIMI III flow (90.1% vs 78%, *p* = 0.019) and corrected TIMI frame count (24 ± 8.43 vs 26.63 ± 9.22, *p* = 0.036) [46].

Cangrelor and glycoprotein IIb/IIIa inhibitors are frequently employed antiplatelet agents when addressing no-reflow scenarios. While the PITRI (Platelet Inhibition to Target Reperfusion Injury) trial centers on evaluating cangrelor’s potential to mitigate the extent of STEMI and microvascular obstruction [47], the utilization of glycoprotein IIb/IIIa inhibitors in no-reflow treatment remains unsupported by existing studies [48]. An illuminating perspective arises from the On-TIME-2 (Ongoing Tirofiban in Myocardial Infarction Evaluation 2) study, which unveiled noteworthy observations. This study underscored that ST-segment elevations, whether prior to PCI (10.9 ± 9.2 mm vs. 12.1 ± 9.4 mm, *p* = 0.028) or an hour post PCI (3.6 ± 4.6 mm vs. 4.8 ± 6.3 mm, *p* = 0.003), exhibited a marked decrease among patients who underwent pretreatment with high-dose tirofiban compared to those receiving placebo [49]. However, the optimal mode of glycoprotein IIb/IIIa inhibitor administration, whether intravenous or intracoronary, remains shrouded in uncertainty.

Indeed, the CICERO (Comparison of Intracoronary Versus Intravenous Abciximab Administration During Emergency Reperfusion of ST-Segment Elevation Myocardial Infarction) and the INFUSE-AMI (Intracoronary Abciximab and Aspiration Thrombectomy in Patients with Large Anterior Myocardial Infarction) trials demonstrated a more substantial reduction in infarct area when compared to the intravenous route [50, 51]. Specifically, in the CICERO trial, myocardial blush grade (i.e., a grading of myocardial perfusion based on the contrast density in the myocardial region of the infarct-related artery compared to regions of non-infarct-related arteries on coronary angiography) was more likely to be within normal limits in the intracoronary infusion group compared to the intravenously treated group (76% vs 67%; *p* = 0.022) [50]. Furthermore, the INFUSE-AMI trial spotlighted a significant reduction in the 30-day infarct size of patients treated with intracoronary abciximab, compared to patients not administered abciximab (median: 15.1%; interquartile range: 6.8%–22.7% vs. 17.9%; 10.3%–25.4%; *p* = 0.03) [51]. In contrast, the AIDA STEMI (Abciximab Intracoronary versus intravenous Drug Application in STEMI) study showed that intracoronary rather than intravenous abciximab was associated with a similar frequency of the primary composite outcome, which encompassed all-cause mortality, re-infarction, or congestive heart failure within 90 days of randomization in 1876 patients (7.0% vs 7.6%; odds ratio [OR]: 0.91; 95% CI 0.64–1.28; *p* = 0.58) [50, 51, 52].

### 4.2 Fibrinolysis as an Emerging Therapeutic Approach to No-Reflow

Fibrinolytic therapy is a well-recognized effective treatment for acute coronary thrombosis when timely PCI is not feasible [1]. However, the approach of combining PCI with systematic administration of lytic therapy, known as ‘facilitated PCI’, has proven to be detrimental in both full-dose [53] and half-dose [54] scenarios. While pre-PCI fibrinolysis can enhance the initial patency of the infarct-related artery, it also amplifies the thrombotic burden, leading to a higher incidence of ischemic side effects and major bleedings compared to conventional primary PCI.

In a 2007 pilot study, Sezer *et al*. [55] revealed that intracoronary administration of low-dose streptokinase immediately after primary PCI could potentially dissolve embolic thrombi in both epicardial coronary arteries and microcirculation, resulting in improved myocardial perfusion. The study focused on a subgroup of STEMI patients undergoing primary PCI who were randomly assigned to receive intracoronary 250,000 IU of streptokinase after the procedure, in addition to standard antiplatelet and anticoagulant therapy. Two days after the intervention, the streptokinase-treated group showed a significant improvement in all indices of microcirculatory function, including coronary flow reserve and index of microvascular resistance, when compared to the control group. Importantly, no major TIMI bleeds were documented during the acute phase. Furthermore, the streptokinase group had significant reductions to the left ventricular infarct area, left ventricular volume, and significantly greater left ventricular ejection fraction at 6-month follow-up evaluation compared to the control group [54].

The rationale of the use of a low intracoronary (i.c.) dose (approximately 20% of the systemic dose of fibrinolytic) during PCI is based on the potential of the intracoronary route to limit systemic fibrinogen depletion, potentially decreasing the occurrence of major bleeding complications [55]. Additionally, selective intracoronary administration enables the rapid and precise delivery of the drug at the desired concentration directly to the thrombus site when compared to systemic administration [56].

Following the groundbreaking study by Sezer *et al*. [55], several randomized controlled trials have investigated the feasibility of intracoronary fibrinolysis in STEMI patients undergoing primary PCI [57, 58, 59, 60, 61, 62, 63]. Initially, intracoronary streptokinase and urokinase were employed as fibrinolytic strategies, and later, newer agents such as alteplase and prourokinase were tested [58, 59, 60]. Notably, the DISSOLUTION (Delivery of thrombolytIcs before thrombectomy in patientS with ST-segment elevatiOn myocardiaL infarction Undergoing primary percuTaneous coronary intervention) trial has recently presented its final results [64]. In this trial, 102 patients with STEMI and high thrombotic burden in the infarct-related artery were randomized to receive an intra-thrombus bolus of 200,000 IU of urokinase or saline solution 5 minutes before PCI [64]. The endpoints included the final TIMI flow grade (i.e., an angiographic grading system widely used to assess epicardial coronary blood flow), and TIMI frame count (i.e., an angiographic grading system that assesses perfusion in the capillary bed at the tissue level) [64]. Localized administration of urokinase yielded a significantly higher occurrence of TIMI flow grade 3 (90% vs 66%, *p* = 0.008) and a reduced post-PCI TIMI frame count (19 ± 15 vs 25 ± 17, *p* = 0.033) [64]. Moreover, at the 6-month follow-up, patients treated with urokinase demonstrated improved survival without major adverse cardiac events (6% vs 21%; log-rank *p* = 0.044) [64]. Notably, at the 1-year echocardiography assessment, urokinase-treated patients displayed a notably diminished left ventricular size (left ventricular end-diastolic volume: 107 ± 38 mL with urokinase vs 125 ± 35 with placebo, *p* = 0.018), an elevated left ventricular ejection fraction (51 ± 14% with urokinase vs 46 ± 10% with placebo, *p* = 0.048), and an improved wall motion score index (1.17 [95% CI: 1.05–1.40] with urokinase vs 1.29 with placebo [95% CI: 1.20–1.51; *p* = 0.021]) [65]. Over a span of 5 years, the urokinase group experienced significantly fewer major cardiac adverse events (10 out of 48 patients vs 22 out of 47 patients, p = 0.023), primarily due to a diminished incidence of hospitalizations for heart failure (3 out of 48 patients vs 11 out of 47 patients, *p* = 0.038) [65].

To date, T-TIME (A Trial of Low-Dose Adjunctive Alteplase During Primary PCI) is the largest study on low-dose fibrinolysis [60]. In this trial 440 patients were randomized to 20-mg alteplase, or 10 mg of alteplase or placebo [60]. Notably, microvascular obstruction did not differ between the 20-mg alteplase and placebo groups (3.5% vs 2.3%; estimated difference, 1.16%; 95% CI: –0.08% to 2.41%; *p* = 0.32) or between the 10-mg alteplase and placebo groups (2.6% vs 2.3%; estimated difference, 0.29%; 95% CI: –0.76% to 1.35%; *p* = 0.74). Major adverse cardiac events (cardiac death, nonfatal myocardial infarction, unplanned hospitalization for heart failure) occurred in 15 patients (10.1%) in the placebo group, 18 (12.9%) in the 10-mg alteplase group, and 12 (8.2%) in the 20-mg alteplase group. Furthermore, there were no differences in major bleed events, which was limited to one patient each from the 10-mg and 20-mg alteplase groups, thus highlighting the safety of the low-dose fibrinolysis strategy [66, 67, 68].

A recent meta-analysis of 13 clinical trials examined randomized STEMI patients receiving low-dose fibrinolysis at time of primary PCI [8]. In this analysis with a total of 1876 patients (Table 1, Ref. [55, 57, 59, 60, 61, 62, 63, 64, 69, 70, 71, 72, 73]), i.c. fibrinolysis was linked to a reduction in major adverse cardiac events (odd ratio: 0.65, *p* = 0.003), and a greater left ventricular ejection fraction at 6-month follow-up (mean difference: 3.78, *p = *0.0010). Additionally, patients who underwent intracoronary fibrinolysis showed significantly better post-PCI corrected TIMI frame count (mean difference = –3.57; *p* < 0.00001), myocardial blush grade 2/3 (odd ratio = 1.76; *p* = 0.008), and normalization of ST elevation (odd ratio: 1.97; *p* = 0.0007). Importantly, the intracoronary fibrinolysis group did not report any significant increase in bleeding events (OR: 1.27; *p* = 0.53). Based on these results, the authors concluded that intracoronary thrombolysis coupled with primary PCI warrants an improvement in major adverse cardiac events and myocardial microcirculatory function in STEMI patients treated with primary PCI which is not associated with any substantial increase in the frequency of major bleeding.

**Table 1.** **Randomized clinical trials on low-dose thrombolytic administration at time of primary percutaneous coronary intervention**.

Study	Type of lytic used	Timing	Dose
Sezer *et al*. [55]	Streptokinase	Post primary PCI (post-IRA recanalization/poststenting)	250 kU
Sezer *et al*. [57]	Streptokinase	Post primary PCI (post-IRA recanalization/poststenting)	250 kU
Greco *et al*. [64]	Urokinase	During primary PCI (intrathrombus)	200 kU
Geng *et al*. [59]	Prourokinase	During primary PCI I (post-IRA balloon dilatation)	10 mg
Fu *et al*. [61]	Prourokinase	During primary PCI (intrathrombus)	10–20 mg
Gibson *et al*. [63]	Alteplase	Post primary PCI (post-IRA recanalization/poststenting	0.3 mg/kg
Ibrahim *et al*. [69]	Alteplase	Post primary PCI (post-IRA recanalization/poststenting)	0.3 mg/kg
Xiao *et al*. [70]	Prourokinase	Post primary PCI (post-IRA recanalization	10–20 mg
McCartney *et al*. [60]	Alteplase	Post primary PCI (post-IRA recanalization	10 and 20 mg
Wang *et al*. [62]	Prourokinase	Post primary PCI (post-IRA thrombus aspiration)	10 and 20 mg
Wu *et al*. [71]	Prourokinase	Post primary PCI (post-IRA recanalization/prestenting)	10 mg
Huang *et al*. [72]	Prourokinase	Post primary PCI (post-IRA recanalization/prestenting)	20 mg
Jiang *et al*. [73]	Prourokinase	Post primary PCI (post-IRA recanalization/prestenting)	10 mg

IRA, infarct-related artery; PCI, percutaneous coronary intervention.

The beneficial effects observed in the majority of low-dose fibrinolysis trials may be dependent on the method of drug delivery. By administering a pharmacologic agent at the site of arterial injury, a reservoir of the drug is created, potentially enhancing the dissolution of intraluminal clots [74]. In addition, lesion-directed delivery of drug may have a mechanical effect that might aid in breaking down the thrombus, thus increasing the surface area for fibrinolysis binding [69]. Overall, the advantages of local vs intracoronary administration might explain at least in part differences in the results between trials. For instance, in the T-TIME trial [60], administration of fibrinolysis was done prior to stent implantation through the manual delivery of drug into the infarct-related artery, whereas, in the DISSOLUTION trial, administration was local [64].

One of the most important advantages of the local use of fibrinolytic drug is improved safety. Gurbel *et al*. [74] demonstrated that intracoronary infusion of alteplase (20 mg) was unable to induce fibrinogen depletion and thrombin activation. Similarly, Sezer *et al*. [57] calculated that a small dose of intracoronary streptokinase achieves a concentration 50 times higher than conventional intravenous administration.

In addition, low-dose intracoronary fibrinolysis exhibits greater pharmacologic activity in both the epicardial coronary arteries and coronary microcirculation. In essence, using a locally delivered low-dose fibrinolytic agent appears to strike the right balance, being sufficiently high to induce effective fibrinolysis while minimizing the risk of increased bleeding.

## 5. Conclusions

No-reflow has been extensively investigated in recent decades, leading to the adoption of several different strategies to manage this common complication. Along with clinical studies testing different therapeutic options, considerable scientific effort has been carried out to identify the multiple pathophysiologic mechanisms that underlie the phenomenon. Notably, histological investigations of acute coronary occlusion have revealed the presence of organized old thrombi rather than fresh thrombi, explaining why several pharmacologic agents or mechanical tools tested during primary PCI have often yielded suboptimal results [22, 23, 24]. On the basis of the histologic findings, a number of randomized controlled trials have recently studied the role of fibrinolysis in STEMI patients undergoing primary PCI.

Current evidence supports the concept that low-dose fibrinolytic therapy coupled with primary PCI may represent an effective strategy to manage the burden of epicardial and microvascular thrombus, and may improve the short and long-term outcome of STEMI. However, preliminary results on low-dose fibrinolysis should be considered hypothesis-generating and therefore further investigations are needed. With this respect, several trials assessing the role of adjunctive fibrinolysis at time of primary PCI are ongoing aiming to identify a personalized approach to management of STEMI. Results of these trials are much awaited in order to elucidate if the effects of low-dose fibrinolysis coupled with primary PCI are different in patients with different ischemic times at presentation [75].
